# Göttingen Minipigs as a Model for Assessing the Impact of Drugs on the Gut and Milk Microbiota—A Preliminary Study

**DOI:** 10.3390/nu16234060

**Published:** 2024-11-26

**Authors:** Silvia Bencivenni, Patrizia Brigidi, Augusta Zannoni, Domenico Ventrella, Alberto Elmi, Maria Laura Bacci, Monica Forni, Federica D’Amico, Silvia Turroni

**Affiliations:** 1 Unit of Microbiome Science and Biotechnology, Department of Pharmacy and Biotechnology, University of Bologna, 40126 Bologna, Italy; silvia.turroni@unibo.it; 2 Department of Veterinary Medical Sciences, University of Bologna, Ozzano dell’Emilia, 40064 Bologna, Italy; augusta.zannoni@unibo.it (A.Z.); domenico.ventrella2@unibo.it (D.V.); alberto.elmi@unipi.it (A.E.); marialaura.bacci@unibo.it (M.L.B.); 3 Department of Medical and Surgical Sciences, University of Bologna, 40138 Bologna, Italy; patrizia.brigidi@unibo.it (P.B.); monica.forni@unibo.it (M.F.); 4 Health Sciences and Technologies-Interdepartmental Center for Industrial Research (CIRI-SDV), University of Bologna, 40126 Bologna, Italy; 5 Department of Veterinary Sciences, University of Pisa, 56124 Pisa, Italy

**Keywords:** lactation, milk microbiota, Göttingen Minipigs, translational model, amoxicillin

## Abstract

Background: Early gut microbiota (GM) dysbiosis can affect a child’s health and has been linked to the onset of pathologies later in life. Breast milk is recognized as a major driver of the structure and dynamics of an infant’s GM. In addition to nutritious and prebiotic compounds, milk contains a microbiota that is shaped by several maternal factors, including gut microorganisms and medications. However, the impact of the latter on the milk microbiota is still largely unknown. Here, we investigated the effects of amoxicillin on the milk microbiota and GM of lactating Göttingen Minipigs sows, a promising model for studying medication transfer during lactation. Methods: Three sows were given amoxicillin (7 mg/kg/day) for three weeks starting from the second week after farrowing. Fecal and milk samples were collected before and after treatment and profiled by 16S rRNA amplicon sequencing. Results: Göttingen Minipigs’ milk microbiota showed similarities to that of humans and conventional sows, with minor compositional shifts after treatment. At the genus level, we observed a decrease in *Staphylococcus* and *o_Bacteroidales;Other;Other*, and an increasing trend in the abundance of *Streptococcus*, *Stenotrophomonas*, *f_Rhodobacteraceae;Other*, *Proteiniclasticum*, *f_Propionibacteriaceae;Other* and *Gemella*. In contrast, as expected, the GM was strongly affected by amoxicillin, even at the phylum level. Conclusions: In addition to demonstrating the relevance of Göttingen Minipigs as a valid model for studying the impact of medications on maternal milk and GM, our findings suggest that the milk microbiota may be more stable during antibiotic treatment than the GM.

## 1. Introduction

While breast milk is acknowledged as the gold-standard nourishment for infants due to its numerous health benefits [1], a knowledge gap regarding medications’ impact on maternal milk still exists. As a result, women receiving postpartum medications for chronic conditions or other health issues, such as infections and mastitis, are often advised to stop breastfeeding or postpone drug treatment, at the risk of compromising their health [2,3]. Developing standardized methodologies for assessing medication safety during lactation is essential to prevent such situations; this is one of the objectives of the IMI ConcePTION program, a European project that aims to reduce uncertainty about the effects of medications used during pregnancy and breastfeeding [4].

After birth, infants start interacting with external microorganisms and this contributes to shaping their gut microbiota (GM). Early disruptions of GM composition and functionality, known as dysbiosis, can have lasting effects on a child’s health and have been associated with an increased risk of developing later-life pathologies such as inflammatory bowel disease, obesity, food allergies, asthma, atopic eczema and many others [5,6]. An infant’s GM assembly is influenced by several factors, including the environment, feeding method, antibiotic exposure, gestational age, type of delivery and vertical transmission of microorganisms from the mother from different body sites (gut, skin, oral cavity, vagina and milk) [7,8,9]. Trough breastfeeding, the microbial communities on the mother’s skin [10] and the microorganisms present in the maternal milk, such as *Staphylococcus*, *Streptococcus*, *Lactobacillus*, *Pseudomonas*, *Bifidobacterium*, *Corynebacterium*, *Enterococcus*, *Acinetobacter*, *Rothia*, *Cutibacterium*, *Veillonella* and *Bacteroides* [11], can seed the newborns’ GM [12] and have a role in the development of the immune system [13]. The most probable sources of the breast milk microbiota are the mother’s skin and the infant’s mouth through the mammary ducts and the mother’s gut through the entero-mammary pathway [10]. This implies that the alteration of the maternal GM could influence which bacteria are translocated to the mammary gland. Breast milk also contains prebiotic compounds, such as human milk oligosaccharides, that foster the growth of health-promoting microorganisms in the infant’s gut [14]. However, current non-clinical models for assessing drug transfer during lactation [15] fall short in evaluating the effects of medications on the milk microbiota. In this context, the Göttingen Minipigs [16] appear to be a promising way to study changes in the microbiota. In the review by Ventrella et al. (2021), Göttingen Minipigs are suggested as an optimal choice due to their physiological and metabolic similarities to humans, highly standardized background, defined microbiology status [17] and ease of handling due to their small size. The volume of milk produced by minipigs is larger compared to rodents and their use in research is more accepted by society as opposed to dogs and non-human primates [16]. Furthermore, although the conventional duration of lactation in pigs is relatively short (approximately 28 days) compared to in humans, it still captures all the different phases of this process, including the dynamic development of the mammary gland. Finally, food reward-based training protocols can be implemented to allow for relatively easy milk sampling, without disturbing the nursing process.

Here, we aimed to study the impact of medications on the milk microbiota in the framework of the IMI Conception project, setting up a comprehensive, wider model of lactation transfer in Göttingen Minipigs. For this purpose, amoxicillin was chosen as the first test molecule. Amoxicillin is a beta-lactam antimicrobial drug effective against a wide range of pathogens, such as *Streptococcus*, *Staphylococcus*, *Pneumococcus* and *Clostridium* species, *Haemophylus influenzae*, *Helicobacter pylori* and *Listeria monocytogenes*, some *Escherichia coli* strains, and *Enterococcus*, *Salmonella* and *Shigella* spp. [18]. Amoxicillin is an acceptable antibiotic in nursing mothers [19] and it is also widely used in pigs to treat systemic infections caused by *Streptococcus suis*, *Haemophilus parasuis*, *Actinobacillus suis* and *Erysipelothrix rhusiopathiae;* respiratory infections caused by *Pasteurella multocida*, *Actinobacillus pleuropneumoniae* and *Bordetella bronchiseptica*; and enteric infections caused by *Escherichia coli* and *Salmonella* spp. [20]. Moreover, amoxicillin has known effects on the GM [21]. In addition, since the gut bacterial community can influence the milk microbiota composition, we also investigated the maternal GM.

## 2. Materials and Methods

### 2.1. Animals

Three pregnant Göttingen Minipigs sows (I19, I20, I21) were purchased from Ellegaard Göttingen Minipigs A/S (Soroe Landevej 302, 4261, Dalmose, Denmark) and transferred to the porcine experimental facility of the Department of Veterinary Medical Sciences of the University of Bologna (Via Tolara di Sopra 50, Ozzano dell’Emilia, 40064, BO, Italy) one month before the expected farrowing date. On arrival, the sows were 22 ± 2 months old, with at least 2 previous successful pregnancies and an average weight of 49 kg. The health status of the provider’s colonies is checked twice a year according to the FELASA recommendations, and the sows were free of the key porcine pathogens. The animals were trained every day at the provider’s facility and then at the experimental facility, using a dedicated food-reward training protocol, to get accustomed to staff touching their mammary gland area. Training sessions were short (5–10 min), with the aim of not losing the animals’ interest and focus, and mainly consisted of letting sows out of their pen and rewarding them for each positive interaction with the operator, including allowing them to rub their abdomen and touch their nipples. In addition, general health and welfare was checked twice a day by experienced veterinary medical professionals. Pregnancy was monitored weekly by means of trans-abdominal ultrasound scans. The sows were fed a standard diet suitable for their breed (Basic Micropigs 9AB17 by Mucedola s.r.l., Settimo Milanese, 20019, MI, Italy) and given water ad libitum. The animals were housed at 21 ± 1 °C with a 12/12 light/dark cycle. One week prior to the expected farrowing day, the sows were moved into customed farrowing pens, with nesting material such as hay.

### 2.2. Experimental Design and Sampling

The present study relies on opportunistic samplings performed during a wider trial aimed at assessing drug exposure among offspring via milk during lactation using amoxicillin as a test compound. The detailed study design is described by Ventrella D. (2022) [22] and was approved by authorization n° 32/2021-PR (Risp. a prot. 2216A.17) of the ethics committee and the Italian Ministry of Health as per local and European regulations.

**Figure 1:**
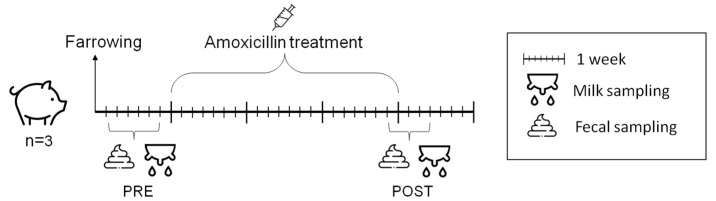
Experimental design. Amoxicillin treatment (7 mg/kg/day) started one week after farrowing and continued for three weeks. Stool and milk samples were collected before trial onset (PRE) and after approximately three weeks of treatment (POST). Picture created with icons from “https://www.freepik.com/ (accessed on 20 November 2024)”.

The trial began one week after farrowing. Sows were dosed intramuscularly with amoxicillin (Clamoxyl^®^ RTU by Zoetis, Rome, Italy) at 7 mg/kg/day for the next 3 weeks until piglet weaning. Stool and milk samples were collected after farrowing, before the trial onset (PRE) and after approximately three weeks of treatment (POST) (Figure 1). To stimulate milk ejection, the sows were administered with oxytocin (Neurofisin by FATRO, Ozzano dell’Emilia, BO, Italy; 10–20 IU, IM), and milk was manually collected, after wiping the mammary gland area and nipples with sterile saline, into sterile conic tubes more than once a day and then pooled. Despite wearing gloves, operators avoided coming into direct contact with the milk by only touching the more proximal portion of the nipples. The volume of collected milk per nursing session ranged between 500 and 2500 µL. Milk samples were immediately placed on ice, aliquoted into sterile cryo-tubes and stored at −80 °C ± 2 within 5 min. Milk levels of amoxicillin throughout the trial ranged between 0.01 and 2.58 µg/mL [22]. As for fecal samples, the sows were monitored by the operators after being fed, and the samples were collected from the pen floor immediately after defecation. The stools were collected into sterile tubes and frozen within 5 min. Subsequently, to avoid environmental contamination, only the inner part of the stool was used for analysis.

### 2.3. Microbial DNA Extraction

Microbial DNA was extracted from feces and milk using protocols previously described [23], with minor modifications. Briefly, fecal samples (250–300 mg) were suspended in 1 mL of lysis buffer (500 mM NaCl, 50 mM Tris-HCl pH 8, 50 mM EDTA and 4% SDS) with the addition of four glass beads (3 mm diameter) and 0.5 g zirconia beads (0.1 mm diameter) (BioSpec Products, Bartlesville, OK, USA). The samples were then processed three times in a FastPrep-24 instrument (MP Biomedicals, Irvine, CA, USA) at 5.5 movements per s for 1 min and incubated at 95 °C for 15 min. The samples were centrifuged at 15,000× *g* for 5 min at 4 °C; then, the supernatant was added to 260 µL of 10 M ammonium acetate and incubated in ice for 5 min. Solid particles were pelleted by centrifugation at 15,000× *g* for 10 min at 4 °C, and then 1 volume of isopropanol was added to the supernatant. After 30 min of incubation in ice, the samples were centrifuged at 15,000× *g* for 15 min at 4 °C, and the pellet was washed with 70% ethanol and suspended in 100 µL of TE buffer (10 mM TrisHCl, 1mM EDTA pH 8.0). The DNA was then purified using the DNeasy Blood & Tissue kit (Qiagen, Hilden, Germany), following the manufacturer’s instructions. A total of 1 ml of milk was centrifuged at 19,000× *g* for 10 min at 4 °C to remove the protein supernatant and the fat layer. The same protocol for microbial DNA extraction from feces was then applied, with final DNA elution in 100 µL of AE Buffer. For all samples, DNA concentration and quality were assessed using a NanoDrop ND-1000 spectrophotometer (NanoDrop Technologies, Wilmington, DE, USA).

### 2.4. 16S rRNA Amplification and Sequencing

For fecal samples, the V3–V4 hypervariable region of the 16S rRNA gene was amplified using the “16S Metagenomic Sequencing Library Preparation” protocol (Illumina, San Diego, CA, USA) and the 341F and 785R primers with linked Illumina adapter overhang sequences [24]. The indexed and purified amplicon libraries were pooled at 4 nM, denatured and diluted to 6 pM before loading onto the MiSeq flow cell for sequencing on an Illumina MiSeq platform. A 2 × 250 bp paired-end protocol was used according to the manufacturer’s instructions (Illumina).

For milk samples, to avoid non-specific products, 16S rRNA amplicons were recovered from 2% low-melting agarose gels using the NucleoSpin^®^ Gel and PCR clean-up kit (Macherey-Nagel GmbH & Co. KG, Düren, Germany), and then the protocol continued as described above.

### 2.5. Bioinformatics and Statistical Analysis

Raw sequences were processed using PANDAseq [25] and QIIME 2 [26]. Amplicon sequence variants (ASVs) were generated using DADA2 [27]. Taxonomic assignment was performed using the VSEARCH algorithm [28] against the SILVA database version 138.1 [29]. The metrics Faith’s phylogenetic diversity, observed features and Shannon entropy were used to determine richness and evenness (alpha diversity). Beta diversity was assessed using weighted and unweighted UniFrac distances, which were used to construct Principal Coordinate Analysis (PCoA) plots. Statistical analyses were performed using R software (version 4.2.2; R Core Team 2022) and the packages vegan [30] and Made4 [31]. Briefly, data separation in PCoA was tested using PERMANOVA (function “adonis” of vegan). The Wilcoxon signed-rank test was used to assess pre–post differences in alpha diversity and relative taxon abundance. A *p*-value ≤ 0.05 was considered significant, whereas a *p*-value < 0.2 was considered a trend. SourceTracker 1.0.1 was used to estimate the proportion of fecal ASVs contributing to milk ASVs [32].

## 3. Results

### 3.1. Milk Microbiota Diversity and Composition

A total of 154,773 high-quality reads were obtained with a mean of 25,795.5 ± 6885.4 per sample (mean ± SD). The reads were clustered into 2454 ASVs.

No change was observed in the alpha diversity of the milk microbiota after amoxicillin treatment; beta diversity calculated on weighted and unweighted UniFrac distances showed no segregation between communities (Supplementary Materials, Figure S1).

**Figure 2:**
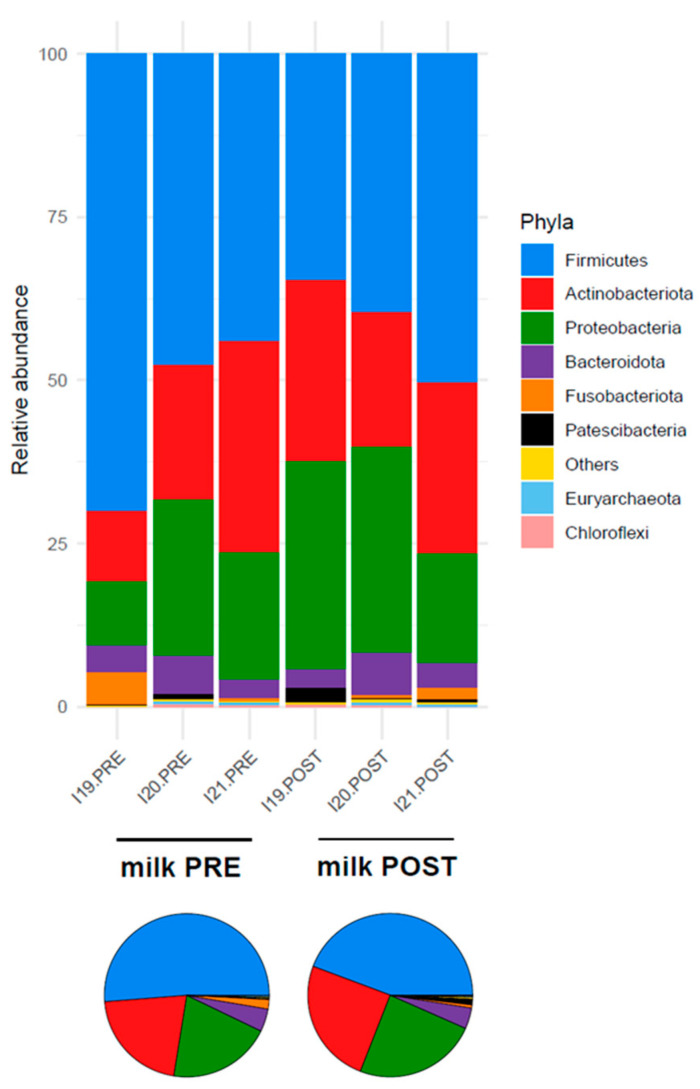
The relative abundance of the major phyla in the milk microbiota of Göttingen Minipigs sows before (PRE) and after (POST) treatment with amoxicillin. The relative abundance of the major phyla in each sample (stacked bar chart) and averaged by time-point (pie charts). Only taxa with a relative abundance > 0.1% in more than 2 samples are shown; the remaining taxa are grouped in “Others”. *N* = 3 for each sample group.

At the phylum level, the pre-treatment milk microbiota was dominated by Firmicutes (relative abundance, mean ± SEM, 54.0% ± 8.2%), followed by Actinobacteriota (21.2% ± 6.3%), Proteobacteria (17.8% ± 4.2%) and Bacteroidota (4.3% ± 0.8%) (Figure 2). No differences were found after amoxicillin administration (*p* > 0.2, Wilcoxon signed-rank test).

**Figure 3:**
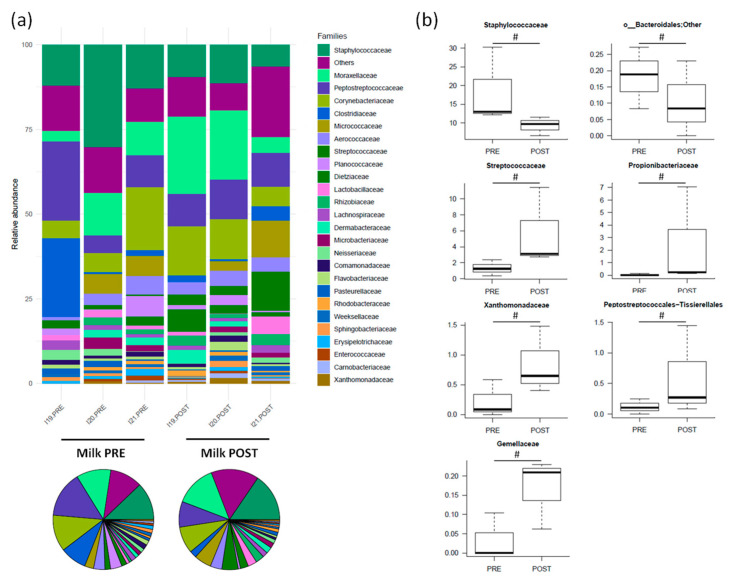
The relative abundance of the major families in the milk microbiota of Göttingen Minipigs sows before (PRE) and after (POST) treatment with amoxicillin. (**a**) The relative abundance of the major families for each sample (stacked bar chart) and averaged by time-point (pie charts). Only taxa with a relative abundance > 0.5% in more than 2 samples are shown; the remaining taxa are grouped in “Others”. (**b**) Boxplots showing the relative abundance distribution of differentially represented families between groups (only taxa with a relative abundance > 0.1% in more than 2 samples are shown) (# *p* < 0.2, Wilcoxon signed-rank test). *N* = 3 for each sample group.

Before treatment, the main families represented were *Staphylococcaceae* (18.5% ± 5.9%), *Peptostreptococcaceae* (12.7% ± 5.5%), *Corynebacteriaceae* (9.8% ± 4.4%), *Clostridiaceae* (8.5% ± 7.4%) and *Moraxellaceae* (8.4% ± 2.8%) (Figure 3a). After amoxicillin administration, *Staphylococcaceae* and *o__Bacteroidales;Other* showed a decreasing trend, while *Streptococcaceae*, *Propionibacteriaceae*, *Xanthomonadaceae*, *Peptostreptococcales-Tissierellales* and *Gemellaceae* tended to increase (*p* < 0.2) (Figure 3b).

**Figure 4:**
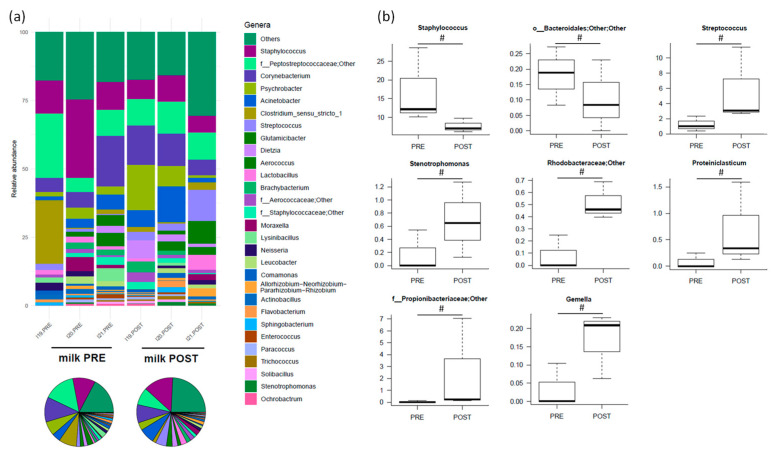
The relative abundance of the major genera in the milk microbiota of Göttingen Minipigs sows before (PRE) and after (POST) treatment with amoxicillin. (**a**) The relative abundance of the major genera for each sample (stacked bar chart) and averaged by time-point (pie charts). Only taxa with a relative abundance > 0.5% in more than 2 samples are shown; the remaining taxa are grouped in “Others”. (**b**) Boxplots showing the relative abundance distribution of differentially represented genera between groups (only taxa with a relative abundance > 0.1% in more than 2 samples are shown) (# *p* < 0.2, Wilcoxon signed-rank test). *N* = 3 for each sample group.

The most relatively abundant genera before treatment were *Staphylococcus* (17.0% ± 5.9%), *f_Peptostreptococcaceae;Other* (12.7% ± 5.5%), *Corynebacterium* (9.8% ± 4.4%), *Clostridium_sensu_stricto_1* (8.4% ± 7.4%) and *Acinetobacter* (3.4% ± 1.2%) (Figure 4a). After amoxicillin administration, *Staphylococcus* and *o_Bacteroidales;Other;Other* tended to decrease, while *Streptococcus*, *Stenotrophomonas*, *f_Rhodobacteraceae;Other*, *Proteiniclasticum*, *f_Propionibacteriaceae;Other* and *Gemella* tended to increase (*p* < 0.2) (Figure 4b).

### 3.2. Gut Microbiota Diversity and Composition

A total of 548,411 high-quality reads were obtained with a mean of 91,401.8 ± 6763.6 per sample (mean ± SD). The reads were clustered into 2454 ASVs.

After amoxicillin treatment, Faith’s phylogenetic diversity of the sows’ GM tended to decrease (*p* = 0.18), while no changes were detected for observed features and Shannon entropy (Supplementary Materials, Figure S2). Similarly, the PCoA plot based on unweighted UniFrac distances showed a trend toward segregation between PRE and POST samples (*p* = 0.1, PERMANOVA), while the weighted UniFrac-based PCoA plot did not (Supplementary Materials, Figure S2).

**Figure 5:**
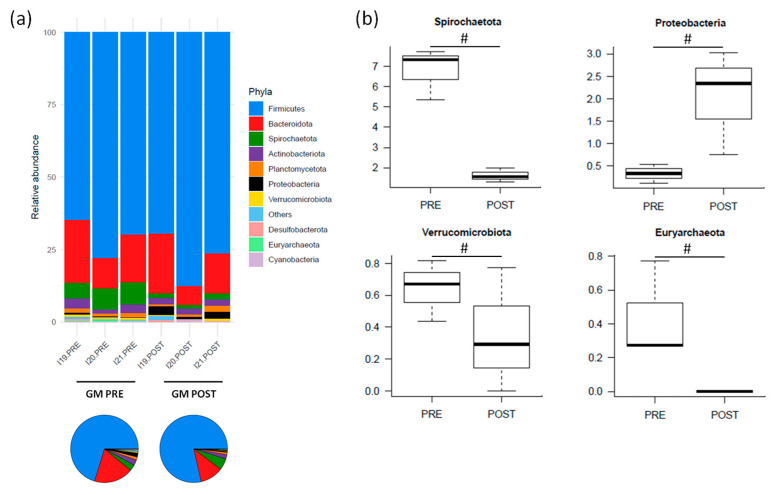
The relative abundance of the major phyla in the gut microbiota (GM) of Göttingen Minipigs sows before (PRE) and after (POST) treatment with amoxicillin. (**a**) The relative abundance of the major phyla for each sample (stacked bar chart) and averaged by time-point (pie charts). (**b**) Boxplots showing the relative abundance distribution of differentially represented phyla between groups (# *p* < 0.2, Wilcoxon signed-rank test). Only taxa with a relative abundance > 0.1% in more than 2 samples are shown; the remaining taxa are grouped in “Others”. *N* = 3 for each sample group.

At the phylum level, the pre-treatment GM was dominated by Firmicutes (relative abundance, mean ± SEM, 70.9% ± 3.9%), followed by Bacteroidota (16.2% ± 3.3%), Spirochaetota (6.8% ± 0.7%) and Actinobacteriota (2.6% ± 0.6%) (Figure 5a). After amoxicillin administration, Spirochaetota, Verrucomicrobiota and Euryarchaeota tended to decrease, while Proteobacteria tended to increase (*p* < 0.2, Wilcoxon signed-rank test) (Figure 5b).

**Figure 6:**
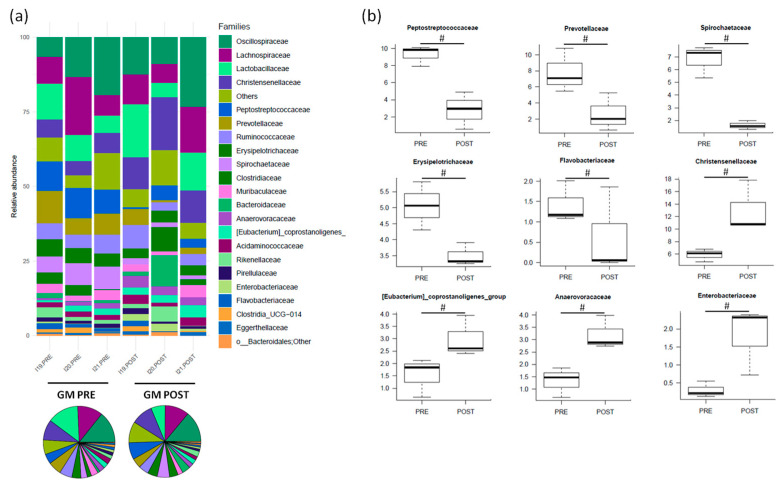
The relative abundance of the major families in the gut microbiota (GM) of Göttingen Minipigs sows before (PRE) and after (POST) treatment with amoxicillin. (**a**) The relative abundance of the major families for each sample (stacked bar chart) and averaged by time-point (pie charts). (**b**) Boxplots showing the relative abundance distribution of differentially represented families between groups (# *p* < 0.2, Wilcoxon signed-rank test). Only taxa with a relative abundance > 0.5% in more than 2 samples are shown; the remaining taxa are grouped in “Others”. *N* = 3 for each sample group.

Before treatment, the main families represented were *Oscillospiraceae* (13.2% ± 3.7%), *Lachnospiraceae* (11.8% ± 3.9%), *Peptostreptococcaceae* (9.3% ± 0.7%), *Lactobacillaceae* (8.8% ± 1.8%), *Prevotellaceae* (7.8% ± 1.6%), *Spirochaetaceae* (6.8% ± 0.7%) and *Christensenellaceae* (5.9% ± 0.6%) (Figure 6a). After amoxicillin administration, *Peptostreptococcaceae*, *Prevotellaceae*, *Spirochaetaceae*, *Erysipelotrichaceae*, *Flavobacteriaceae*, *Bifidobacteriaceae*, *Methanobacteriaceae* and *UCG-010* showed a decreasing trend, while *Christensenellaceae*, *[Eubacterium]_coprostanoligenes_group*, *Anaerovoracaceae*, *Enterobacteriaceae*, *Coriobacteriaceae*, *p_Firmicutes;Other;Other;Other* and *Paludibacteraceae* tended to increase (*p* < 0.2) (Figure 6b; see also Supplementary Materials, Figure S3 for changes in subdominant families).

**Figure 7:**
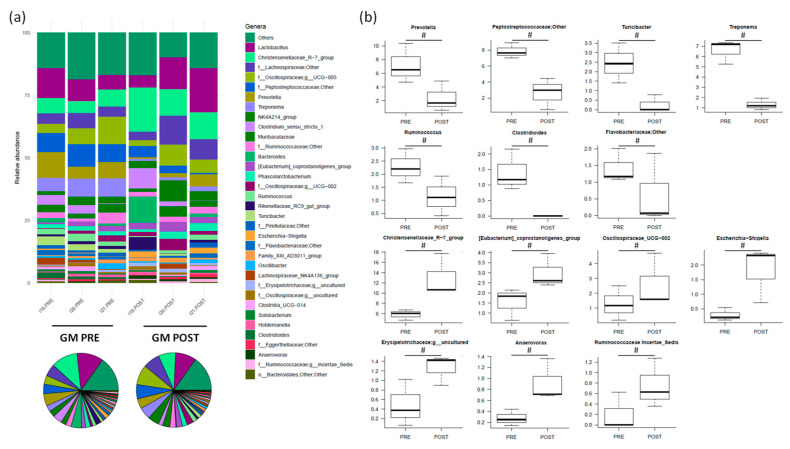
The relative abundance of the major genera in the gut microbiota (GM) of Göttingen Minipigs sows before (PRE) and after (POST) treatment with amoxicillin. (**a**) The relative abundance of the major genera for each sample (stacked bar chart) and averaged by time-point (pie charts). (**b**) Boxplots showing the relative abundance distribution of differentially represented genera between groups (# *p* < 0.2, Wilcoxon signed-rank test). Only taxa with a relative abundance > 0.5% in more than 2 samples are shown; the remaining taxa are grouped in “Others”. *N* = 3 for each sample group.

The most abundant genus before treatment was *Lactobacillus* (8.8% ± 1.8%), followed by *Peptostreptococcaceae;Other* (7.9% ± 0.6%), *Prevotella* (7.2% ± 1.7%) and *Oscillospiraceae UCG-005* (7.0% ± 2.2%) (Figure 7a). After amoxicillin administration, 14 genera (including *Prevotella*, *Peptostreptococcaceae;Other*, *Turicibacter*, *Treponema*, *Ruminococcus*, *Clostridioides* and *Flavobacteriaceae; Other*) showed a tendency to decrease (*p* < 0.2). On the other hand, the relative abundance of 12 families (among which were *Christensenellaceae_R-7_group*, *[Eubacterium]_coprostanoligenes_group*, *Oscillospiraceae UCG-002*, *Escherichia-Shigella*, *Erysipelotrichaceae;g_uncultured*, *Anaerovorax* and *Ruminococcaceae Incertae_Sedis*) slightly increased (*p* < 0.2) (Figure 7b; see also Supplementary Materials, Figure S4 for changes in subdominant genera).

SourceTracker identified an average of 0.10% fecal ASVs in the milk samples before amoxicillin treatment and 0.11% after treatment (Supplementary Materials, Figure S5).

## 4. Discussion

This study aimed to evaluate the impact of amoxicillin on the maternal milk microbiota and GM of Göttingen Minipigs sows, with the additional purpose of assessing the validity of this animal model for studying medication effects on the gut and milk microbiota. Diversity and compositional analysis indicated that the milk microbiota remained overall more stable than the GM during amoxicillin administration.

To the best of our knowledge, the milk microbiota of Göttingen Minipigs has not yet been studied. Chen et al. (2018) investigated the milk microbiota composition of Large-White × Landrace sows [33]. They identified Firmicutes and Proteobacteria as the most abundant bacterial phyla in sow milk, as we found in our study, but the relative abundance of Actinobacteriota was higher in the milk of Göttingen Minipigs. In addition, we detected some low-abundance bacteria (Patescibacteria and Chloroflexi) that were not shared with Large-White × Landrace sows. At the genus level, most core taxa were shared with conventional pigs, such as *Streptococcus*, *Lactobacillus*, *Acinetobacter*, *Moraxella*, *Neisseria* and *Corynebacterium* [33], but *Staphylococcus*, the most represented genus in Göttingen Minipigs milk, was notably under-represented. The Göttingen Minipigs’ milk microbiota also showed some similarities with the human milk microbiota, especially in terms of the dominance of Firmicutes and Proteobacteria, followed by Bacteroidetes [34,35]. Only some studies reported a higher relative abundance of Actinobacteria, close to the proportion found in the Göttingen Minipigs’ milk [36,37]. Furthermore, both *Staphylococcus* and *Streptococcus* are commonly found in human milk as well as *Corynebacterium*, *Flavobacterium*, *Lactobacillus* and *Stenotrophomonas* [35,38]; other genera, such as *Clostridium_sensu_stricto_1* [39] and *Acinetobacter* [37], have also been reported.

Few taxa in milk samples were found to be altered after antibiotic treatment, suggesting that amoxicillin had a limited effect on the milk microbiota and therefore that breastfeeding could be considered safe during maternal treatment. These changes included a rise in the abundance of *Streptococcaceae* and *Streptococcus*, an increase that could be related to antibiotic resistance [40]. The family *Xanthomonadaceae* and its genus *Stenotrophomonas* also increased; however, it should be noted that DNA from *Stenotrophomonas* is commonly present in molecular biology reagents, solutions and kits, so its detection in milk could be a technical artifact [41]. Finally, we found a decrease in *Staphylococcaceae* and *Staphylococcus*; however, their variation is possibly related to lactation time [33] and cannot be attributed with certainty to amoxicillin.

The GM composition of Göttingen Minipigs resembled that of conventional pigs [42], with which they share the most abundant core genera [8,43]. Except for Spirochaetes, the phylum-level GM profile of Göttingen Minipigs was also similar to that of humans [44]. With regard to the effects of amoxicillin, we found a reduction in alpha diversity, which is a common signature of antibiotic treatment [21], and several compositional changes, although without statistical significance. This is most likely due to the small number of animals included in the study. Among the variations, it is worth noting the increase in *Escherichia-Shigella*, which could be related to the intrinsic resistance of *Escherichia coli* to amoxicillin [20]. Based on SourceTracker analysis, a very low percentage of fecal ASVs were found in milk, suggesting the limited impact of the GM on the milk microbiota.

The main limitations of this study are as follows: (1) the small size of the study, which, however, given the high uniformity of the animals used, adequately meets the ethical demands imposed by the 3Rs rule; (2) the lack of collection of feces from piglets, which would have widened the aim of the research to the assessment of drug impact on the assembly of the newborn GM; (3) the lack of consideration of the potential effects of sows’ diet on the composition of the milk and gut microbiota because this was outside the scope of the project. In any case, to avoid possible uncontrolled diet-related alterations, the diet used throughout the trial period was a standard commercial formulation belonging to a single production batch. Furthermore, the study did not include a control group of sows not receiving amoxicillin, so it cannot be excluded that other factors, such as lactation time, contributed to the observed milk microbiota profiles. It should be noted that there is little information in the literature on the temporal stability of the milk microbiota, which does not allow for mechanistic speculation.

## 5. Conclusions

The milk microbiota of Göttingen Minipigs is relatively similar to that of conventional sows and humans, and overall appears to be more stable than the GM after amoxicillin treatment, suggesting limited effects of amoxicillin on the piglets’ GM. Consequently, it is conceivable that the maternal use of amoxicillin during lactation should not induce dysbiosis in breastfed infants. However, some compositional changes were found that deserve further attention. Regarding this, it should be taken into account that multiple factors, other than drug treatment, may be associated with variations in the human milk microbiome, including maternal nutrient intake, maternal BMI, and delivery mode [45]. Göttingen Minipigs could be considered a suitable model to study the impact of medications on the milk and gut microbiota, but further studies with larger numbers of animals and different medications (including a control group) are needed to consolidate the model and to increase our knowledge of the effects of medications on maternal microbiota. Moreover, piglets should be included in future research to evaluate potential GM alterations that could impact their long-term health, and the viable bacteria present in milk should also be investigated to better understand how the milk microbiota influences a piglet’s GM. No less important, obtaining mechanistic information will be essential for translational implications.

## Data Availability

Sequencing reads were deposited in the National Center for Biotechnology Information Sequence Read Archive (NCBI SRA; BioProject ID PRJNA1175632).

## Supplementary Materials

The following supporting information can be downloaded at: https://www.mdpi.com/article/10.3390/nu16234060/s1, Figure S1: Impact of amoxicillin on milk microbiota diversity of Göttingen Minipigs sows; Figure S2: Impact of amoxicillin on gut microbiota diversity of Göttingen Minipigs sows; Figure S3: Families with altered abundance in gut microbiota (GM) of Göttingen Minipigs sows after treatment with amoxicillin; Figure S4: Genera with altered abundance in gut microbiota (GM) of Göttingen Minipigs sows after treatment with amoxicillin; Figure S5. Source of ASVs detected in milk samples.
