# Supplementary material for: Göttingen Minipigs as a Model for Assessing the Impact of Drugs on the Gut and Milk Microbiota—A Preliminary Study
Source: Nutrients. 2024 Nov 26;16(23):4060. doi: 10.3390/nu16234060 (PMC11643497; doi:10.3390/nu16234060)
Supplement: Supplementary file 1 [file nutrients-16-04060-s001.zip › nutrients-3306526-supplementary.pdf]

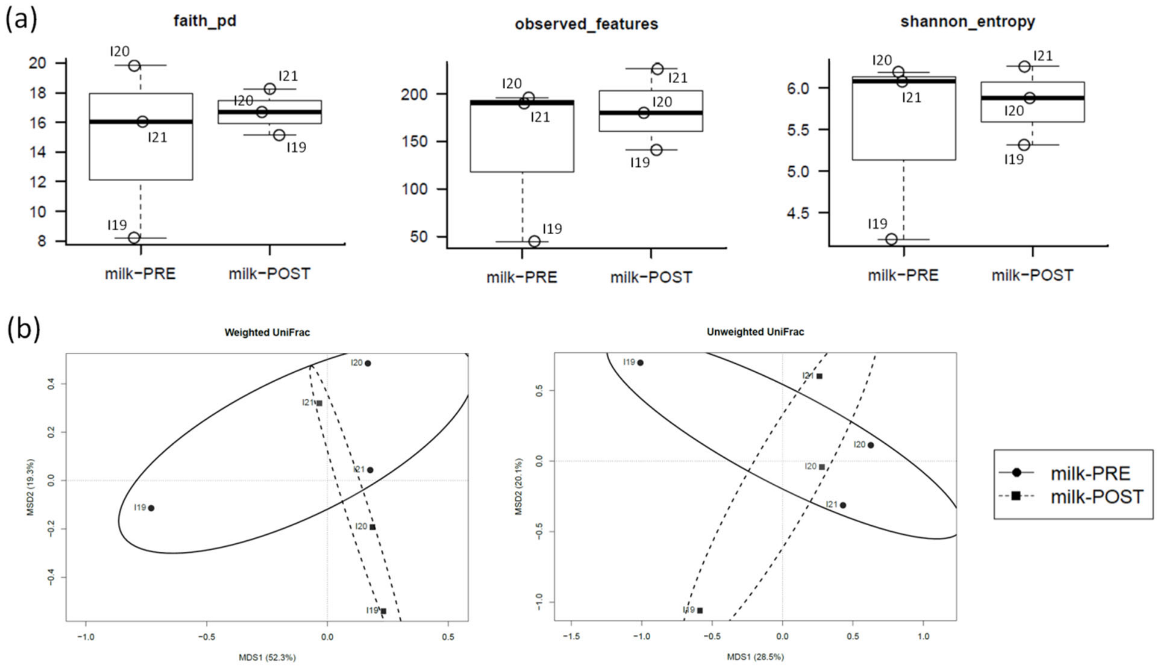


**Figure S1. Impact of amoxicillin on milk microbiota diversity of Göttingen Minipigs sows.** (a) Boxplots showing the distribution of alpha diversity, estimated using Faith's phylogenetic diversity, observed features and Shannon entropy in the gut microbiota of Göttingen Minipigs sows before (PRE) and after (POST) three weeks of amoxicillin treatment (p>0.2; Wilcoxon signed-rank test. (b) Principal Coordinates Analysis (PCoA) plots based on unweighted and weighted UniFrac distances between groups. PERMANOVA, p=0.8 and p=0.8 respectively. *N*=3 for each sample group.

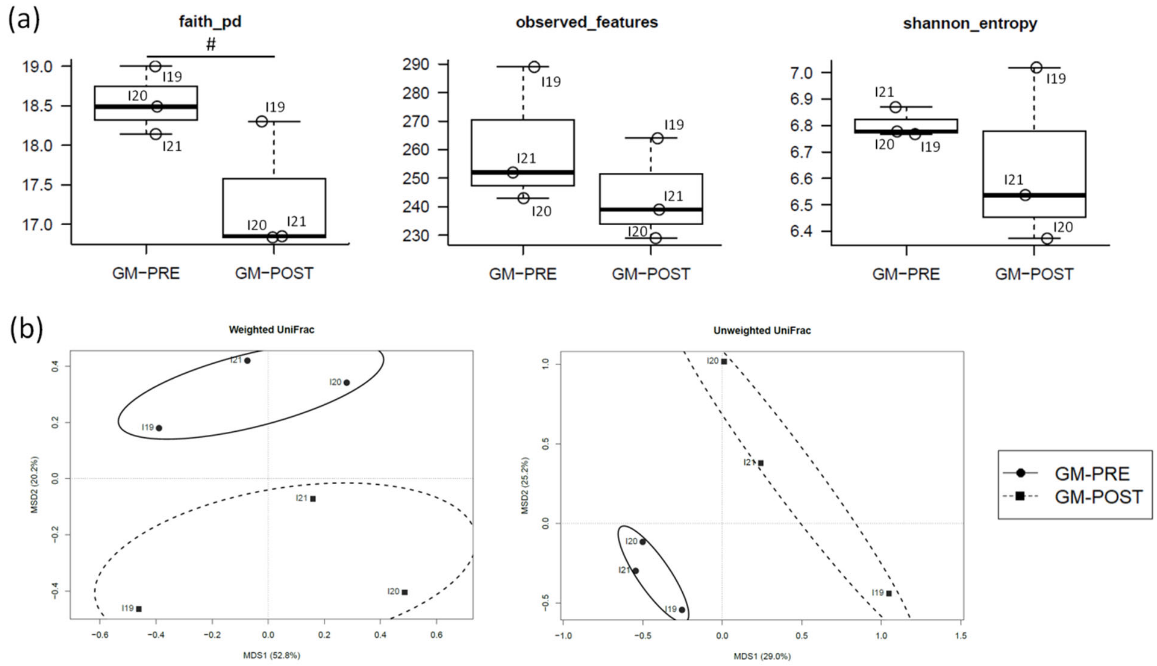


**Figure S2. Impact of amoxicillin on gut microbiota diversity of Göttingen Minipigs sows.** (a) Boxplots showing the distribution of alpha diversity, estimated using Faith's phylogenetic diversity, observed features and Shannon entropy in the gut microbiota (GM) of Göttingen Minipigs sows before (PRE) and after (POST) three weeks of amoxicillin treatment. Wilcoxon signed-rank test, #p<0.2. (b) Principal Coordinates Analysis (PCoA) plots based on weighted and unweighted UniFrac distances between groups. PERMANOVA, p=0.4 and p=0.1 respectively. *N*=3 for each sample group.

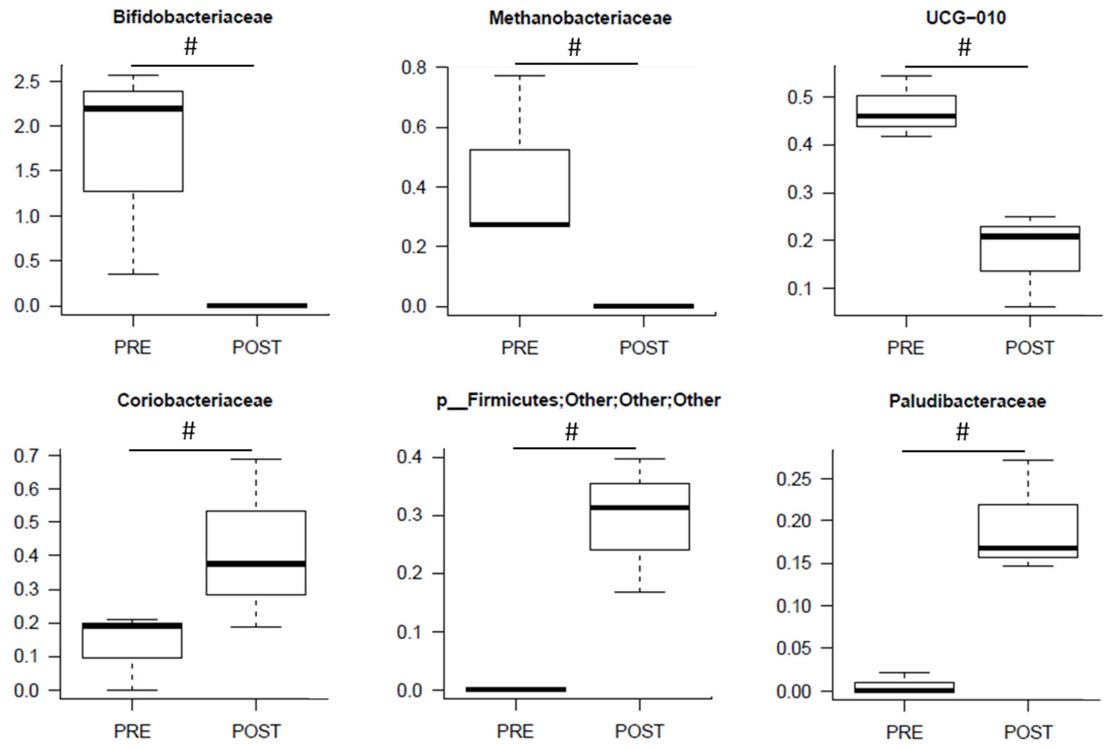


**Figure S3. Families with altered abundance in the gut microbiota (GM) of Göttingen Minipigs sows after treatment with amoxicillin**. Boxplots showing the relative abundance distributions of differentially represented families between groups (only taxa with a relative abundance >0.1% and <0.5% in more than 2 samples are shown) (#p<0.2, Wilcoxon signed-rank test). *N*=3 for each sample group.

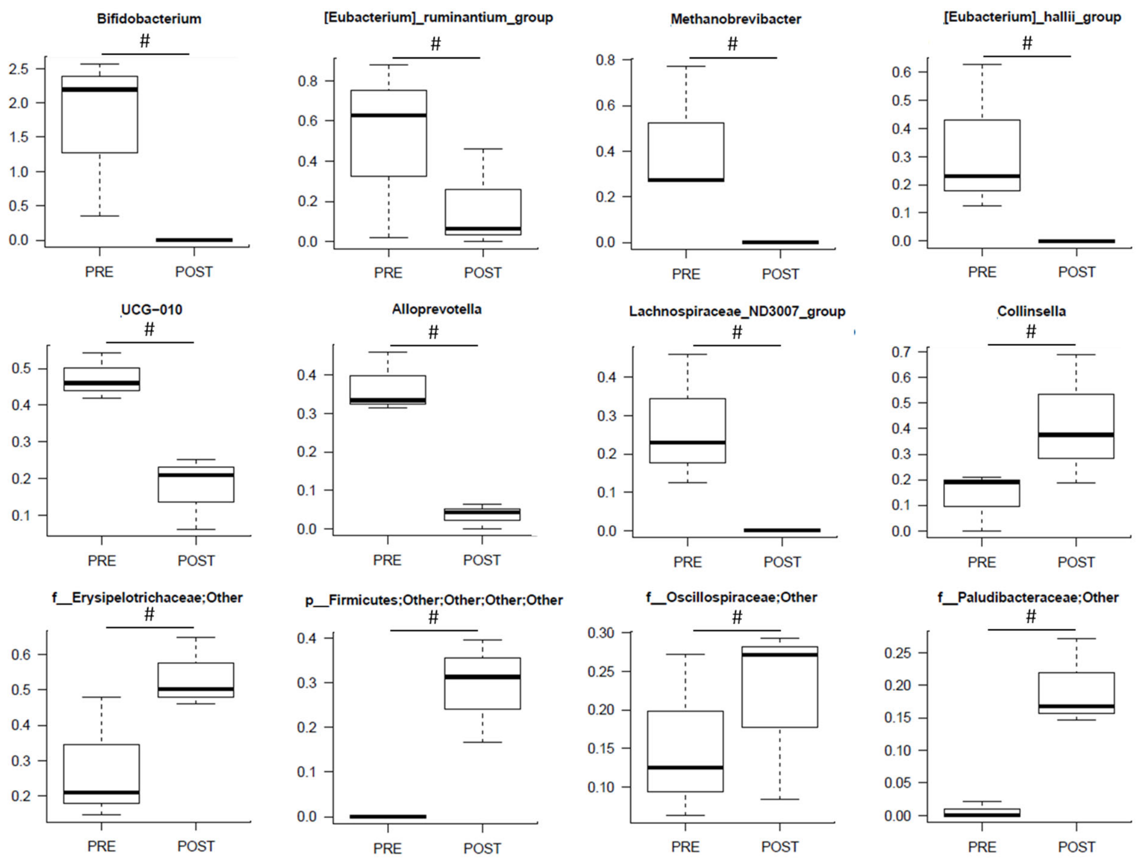


**Figure S4. Genera with altered abundance in the gut microbiota (GM) of Göttingen Minipigs sows after treatment with amoxicillin**. Boxplots showing the relative abundance distributions of differentially represented genera between groups (only genera with a relative abundance >0.1% and <0.5% in more than 2 samples are shown) (#p<0.2, Wilcoxon signed-rank test). *N*=3 for each sample group.

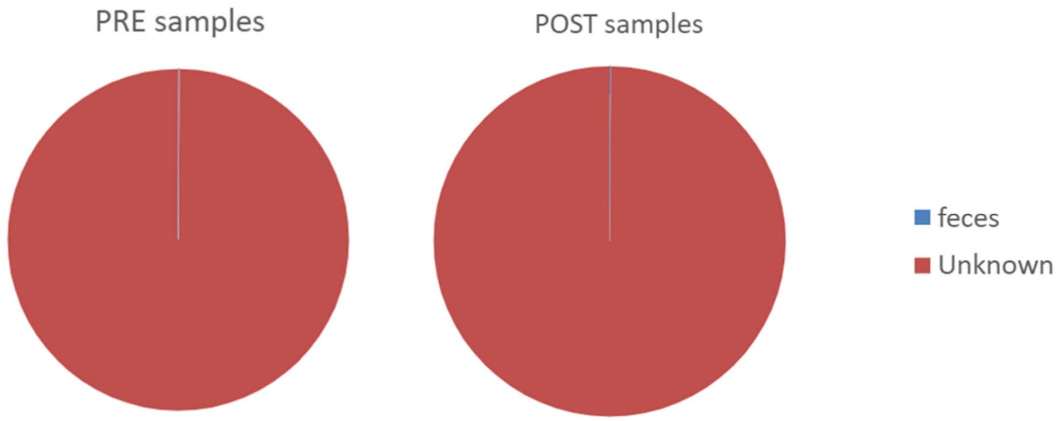


**Figure S5. Source of ASVs detected in milk samples.** Pie chart of the source of ASVs in milk predicted by SourceTracker before (PRE) and after (POST) amoxicillin treatment.
